# The association between measures of static foot posture and foot mobility and foot kinematics during barefoot walking

**DOI:** 10.1186/1757-1146-8-S2-O4

**Published:** 2015-09-22

**Authors:** Andrew K Buldt, George S Murley, Pazit Levinger, Hylton B Menz, Christopher J Nester, Karl B Landorf

**Affiliations:** 1 Department of Podiatry, Faculty of Health Sciences, La Trobe University, Bundoora, VIC, 3086, Australia; 2 Lower Extremity and Gait Studies Program, La Trobe University, Bundoora, VIC, 3086, Australia; 3 Institute of Sport, Exercise and Active Living, Victoria University, Footscray, VIC, 8001, Australia; 4 School of Health Sciences, University of Salford, UK

**Keywords:** Foot, Posture, Biomechanics

## Background

Variations in foot posture are associated with common lower limb injuries. However, there is uncertainty as to which measure of foot posture is the most valid in terms of predicting 3-dimensional kinematics of the foot. The aim of this study was to investigate the association between measures of static foot posture or foot mobility and kinematic variables of the foot during barefoot walking.

## Methods

The foot posture and mobility of 97 healthy adults (46 males, 51 females; aged 24.4 ± 6.2 years) was assessed using the 6-item Foot Posture Index (FPI), Arch index (AI), normalised navicular height (NNHt) and normalised dorsal arch height (DAH). Foot mobility was evaluated using the Foot Mobility Magnitude (FMM) measure. Co-variate measurements included height, weight, BMI, foot length, truncated foot length, stride length, step length, walking speed and range of motion of the ankle, rearfoot and 1^st^ metatarsophalangeal joint. A five-segment foot model measured tri-planar motion of the rearfoot, midfoot, medial forefoot, lateral forefoot and hallux. Peak and range of motion variables during load acceptance and midstance/propulsion phases of gait were extracted for all segments. Hierarchical regression analyses were conducted, accounting for potential confounding variables.

## Results

The variance in peak kinematic and range of motion variables that was independently explained by foot posture measures were as follows: FPI: 5% to 22%, NNHt 6% to 20%, AI: 7% to 13%, DAH 6% to 8%, FMM: 8%. Of all measures of foot posture and mobility, FPI was retained as a significant predictor variable across more regression models, therefore explaining variance across the most number of kinematic segments. However the amount of variance explained by FPI did not exceed other measures. Overall, static foot posture measures were more strongly associated with kinematic variables than foot mobility measures and. explained more variation in peak kinematic variables compared to range of motion variables.

## Conclusions

Foot posture measures can explain a modest amount of variation in foot kinematics. In the context of this study. the FPI was the strongest predictor of dynamic foot motion as measured by foot kinematics.

